# A new standardized data collection system for brain stereotactic external radiotherapy: the PRE.M.I.S.E project

**DOI:** 10.2144/fsoa-2020-0015

**Published:** 2020-05-27

**Authors:** Silvia Chiesa, Barbara Tolu, Silvia Longo, Barbara Nardiello, Nikola Dino Capocchiano, Federica Rea, Luca Capone, Gerardina Stimato, Roberto Gatta, Alessandro Pacchiarotti, Mariangela Massaccesi, Giuseppe Minniti, Francesco Cellini, Andrea Damiani, Mario Balducci, Piercarlo Gentile, Vincenzo Valentini, Federico Bianciardi

**Affiliations:** 1 UOC di Radioterapia Oncologica, Dipartimento Diagnostica per Immagini, Radioterapia Oncologica ed Ematologia, Fondazione Policlinico Universitario A Gemelli IRCCS, Roma, Italia; 2 Radiation Oncology Unit, University of Pittsburgh Medical Center, Hillman Cancer Center, San Pietro Hospital FBF, Rome, Italy; 3 Università Cattolica del Sacro Cuore, Istituto di Radiologia, Roma, Italia; 4 UOC di Fisica Sanitaria, Dipartimento Diagnostica per Immagini, Radioterapia Oncologica ed Ematologia, Fondazione Policlinico Universitario A Gemelli IRCCS, Roma, Italia; 5 KBO.COM Srl, UCSC spinoff, Roma, Italia

**Keywords:** big data, brain, personalized medicine, predictive model, stereotactic

## Abstract

**Background:**

In recent years, novel radiation therapy techniques have moved clinical practice toward tailored medicine. An essential role is played by the decision support system, which requires a standardization of data collection. The Aim of the Prediction Models In Stereotactic External radiotherapy (PRE.M.I.S.E.) project is the implementation of systems that analyze heterogeneous datasets. This article presents the project design, focusing on brain stereotactic radiotherapy (SRT).

**Materials & methods:**

First, raw ontology was defined by exploiting semiformal languages (block and entity relationship diagrams) and the natural language; then, it was transposed in a Case Report Form, creating a storage system.

**Results:**

More than 130 brain SRT’s variables were selected. The dedicated software Beyond Ontology Awareness (BOA-Web) was set and data collection is ongoing.

**Conclusion:**

The PRE.M.I.S.E. project provides standardized data collection for a specific radiation therapy technique, such as SRT. Future aims are: including other centers and validating an extracranial SRT ontology.

## Lay abstract

Radiotherapy moves clinical practice toward tailored medicine, where a decision support system is essential. The Prediction Models In Stereotactic External radiotherapy (PRE.M.I.S.E) project aims to implement a system that can analyze heterogeneous datasets. This article presents the project design for brain stereotactic radiotherapy (SRT). First, a raw ontology, which is a classification system where uniform and nonambiguous definitions represent each variable and all their relationships, was defined by exploiting semiformal and natural language. It was then it was transposed in a case report form, setting a storage system. More than 130 brain SRT’s variables were selected. The dedicated software BOA-Web (Beyond Ontology Awareness) was set. PRE.M.I.S.E. provides standardized data collection for SRT. Future aims are: including other centers and validating an extracranial SRT ontology.

## Background

Recently, oncological knowledge has grown exponentially in terms of both diagnosis and therapy. Qualitative improvements in different fields, for example, genomics, histology and technology, provide a heterogeneity of data regarding both patient and tumor characteristics. Therefore, a larger amount of different types of data, together with their increased complexity, need to be considered in the decision-making process [1,2]. This process has historically been guided by international guidelines, based on randomized clinical trial evidence that provide a patient’s selection beforehand. Population-based observational studies are recently emerging as a complementary form of research, often named ‘rapid learning healthcare’ (RLHC), which is essential to ensure that clinical trials results can be translated into tangible benefits for the general population [3]. Data collection quality in the RLHC approach can be low as data is frequently collected using different procedures, thus, pooled multicenter research is difficult to perform. Standardized data collection improves the quality of this process, defining variables and the way they should be shared without ambiguity [4].

Data collection standardization methods benefit from the use of a common ontology system. Ontologies are commonly defined as an ‘explicit specification of a conceptualization’ [5]: this, in our specific context, is equivalent to a classification system where uniform and not ambiguous definitions represent each variable and all their relationships. A large heterogeneous database is required to store all the information without knowing beforehand what the research topic would be. From the hypothesis, it is determined what features should be included in the learning effort in order to obtain a predictive model that represents the distribution of the same features and their relationship inside the dataset. Predictive models are the basis of predictive tool implementation; beside the more appealing interactive websites, graphical calculating devices, like nomograms [6,7].

In oncological literature, several experiences have been published regarding decision support system (DSS) implementations in different anatomical sites [1,4,8,9], but a DSS for a specific radiation technique is not available yet. The PREdiction Models In Stereotactic External radiotherapy (PRE.M.I.S.E.) is one of the research projects involved in the ‘umbrella protocol’ [9], which works at facilitating RLHC. The aim of the PRE.M.I.S.E. project is to create a consistent dataset to support the future development of DSSs for stereotactic radiotherapy (SRT), moving toward a ‘shared decision making’ approach. Doctors, together with patients, will be able to evaluate pros and cons of different treatment strategies. On the other hand, clinicians will be able to actively discuss and decide for the best therapeutic intervention, once having assessed all the features to optimize a stereotactic treatment plan.

## Materials & methods

A multidisciplinary team was created with members stemming from the first two centers involved. Physicians, physicists, nurses and therapists were involved in the team. The group planned a set of phases and scheduled periodical meetings to assess the development of each single task: this iterative approach (design-implementation-validation-back to design) helped us in exploiting the synergy of the multiple discipline in our team.

The local ethics committee approved the protocol before patients’ accrual according to the legislation of the country.

Our general workflow was divided into different phases and can be summed up as follows:

- Ontology definition;
- Setting of the storage system;
- Data analysis.

### Ontology definition

To reduce the ambiguity in collecting and analyzing data, the first step was the definition of a clear ontology. Even if an ontology can intuitively be represented using the natural language, this is commonly discouraged, because even if it is the simplest solution for a human to human communication it cannot be easily translated into a formal language to be computed by a computer. The opposite, the immediate development of an ontology through the use of one of the many available formal languages, such as the options provided by the World Wide Web Consortium, is a task that requires a specific training, a multidisciplinary task force and the final result cannot easily be made understandable for non-skilled physicians for review or external validation. This also makes validating the ontology a complex task, as many participants in multicentric studies lack the required training.

The radiation oncology ontology [10] is an example of ontology written in web ontology language for radiotherapy, but the complexity of the language and the implications (in particular for automatic reasoning) are an important barrier for an extended use in a real world scenario. Because of the aforementioned reasons, we adopted the following strategy: a raw ontology was defined, exploiting some semi-formal languages (block diagrams and entity relationship diagrams) and the natural language; then it was transposed in a case report form (CRF). A CRF is a format that can be loaded, parsed and executed by a computer, where each single clinical variable is described in terms of type, admitted values, relation with other variables. Here each variable is framed as an attribute of more general entities (or classes) such as patients, treatments, visits and toxicities. The relations among the entities are also provided with the specification of the cardinalities. Once the ontology has been extensively validated and consolidated by the practice, we will consider an implementation with the World Wide Web Consortium technology, in order to exploit the automatic inference for some minor tasks (e.g., descriptive statistics on the cohort).

In building the ontology, the complexity of the knowledge domain has been separated into three different and distinct layers: the registry level is the most general tier and includes the baseline patient and tumor characteristics (age, gender, ethnicity etc.), which are considered relevant for epidemiological analysis only. The Procedure level comprises treatment information and related toxicities, and the evaluation of outcome in terms of disease-free survival and acute and late toxicities. The final level is the Research level, and includes clinical and imaging information used for in-depth, advanced research projects only.

In order to implement and use the ontology and guide the work of the designated data managers, the team created the CRF according to the format compatible with beyond ontology awareness (BOA) – a research electronic data capture software.

### Setting of the storage system

**Figure 1.**
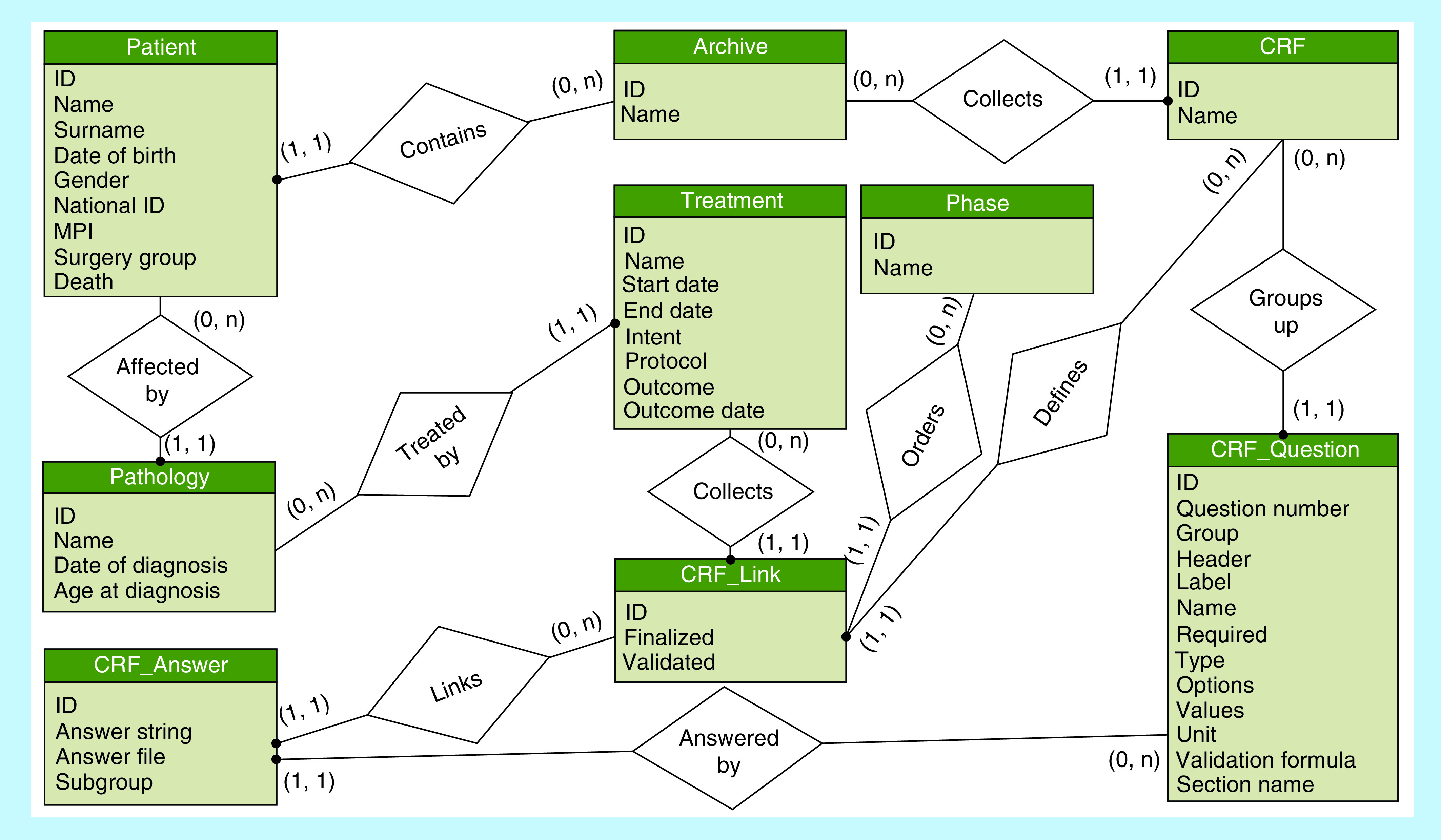
Ontology structure in the SQLite database. CRF: Case report form.

BOA utilizes a relational database model as the base of the data layer. SQLite has been chosen as the designated database in order to guarantee a degree of portability, by allowing installation of the complete service on wide variety of devices. A part of the implemented ontology structured in the SQLite database is shown in Figure 1. Specifically, a single archive was created for the study and successively populated with patients that could have one or more pathologies. Each of these pathologies could have one or more treatments. The specific CRFs designed for this study were then imported into BOA and converted to the required structure, which meant that each CRF had a multitude of related questions of various types (e.g., dates, single-select lists, multiselect lists or other types of inputs) with specific constraints for allowable inputs defined during the definition of the ontology. During the data entry phase, CRF links were automatically generated – linking each recorded answer to a specific question and finally linking each completed CRF link to a specific phase in the patient history (e.g., first contact with the patient). This architecture allows not only to guarantee the integrity of the ontology, but also greatly eases any subsequent data extraction / data analysis effort.

**Figure 2.**
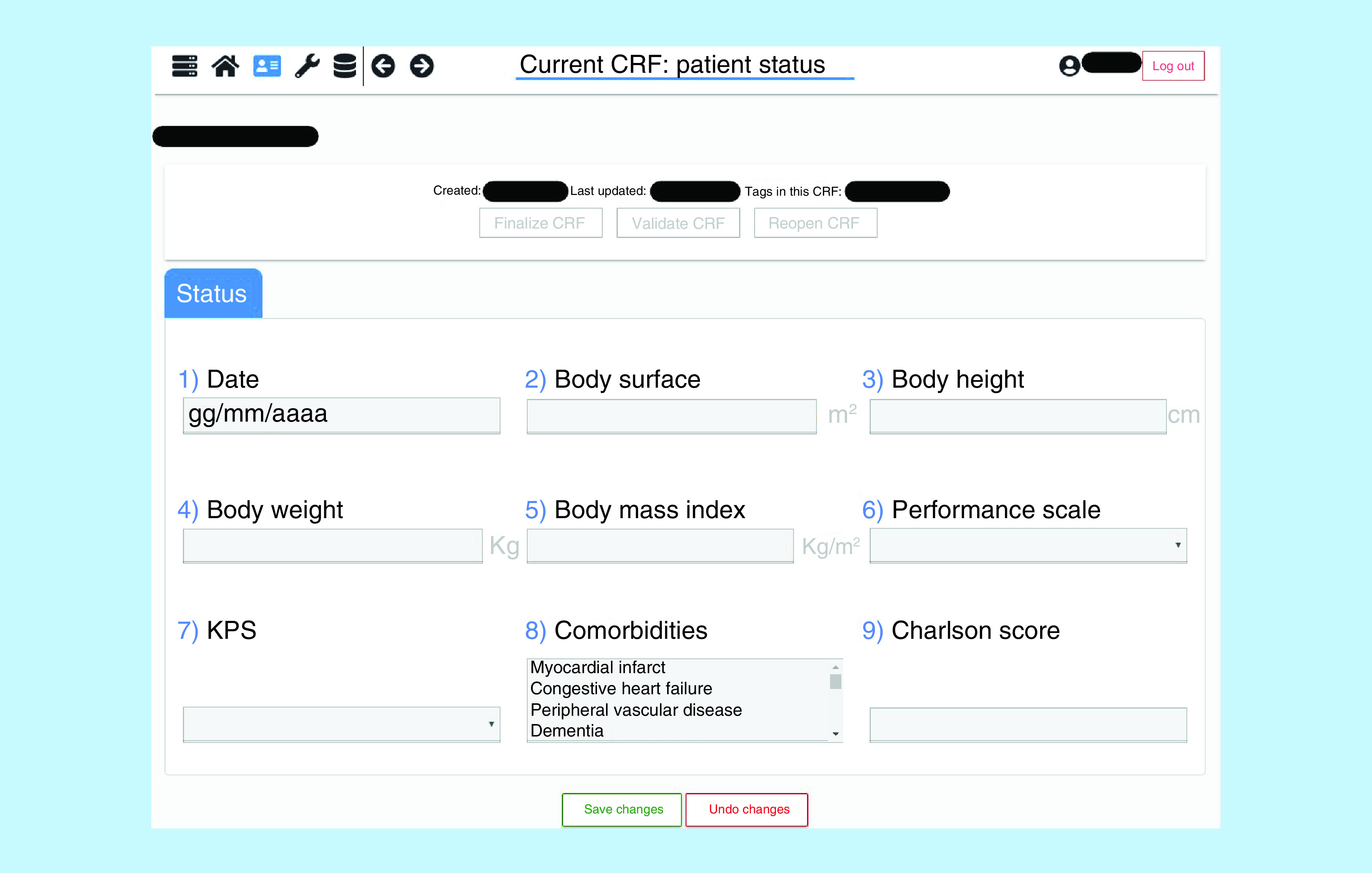
An example screenshot of a case report form. CRF: Case report form.

BOA itself is structured as a portable Django webservice, which allows data managers to quickly access to the required interfaces and automatically handle all required data validation aspects. An example screenshot of a CRF is shown in Figure 2. BOA can store data in two different ways, depending on the needs of the center and the wishes of the participants: BOA.Cloud and BOA.Local.

#### BOA.Cloud

The collected data are automatically anonymized and transferred to a cloud-based large database. After the transfer, it will not be possible to reconstruct the history of the transferred data or the pertaining patient files, due to the complete anonymization algorithm that does not allow identifying information to be conveyed, including unique IDs.

#### BOA.Local

The data are stored in a local database, in a secure area that prohibits any data exchange between the local client and other computers in the local area network or internet.

**Figure 3.**
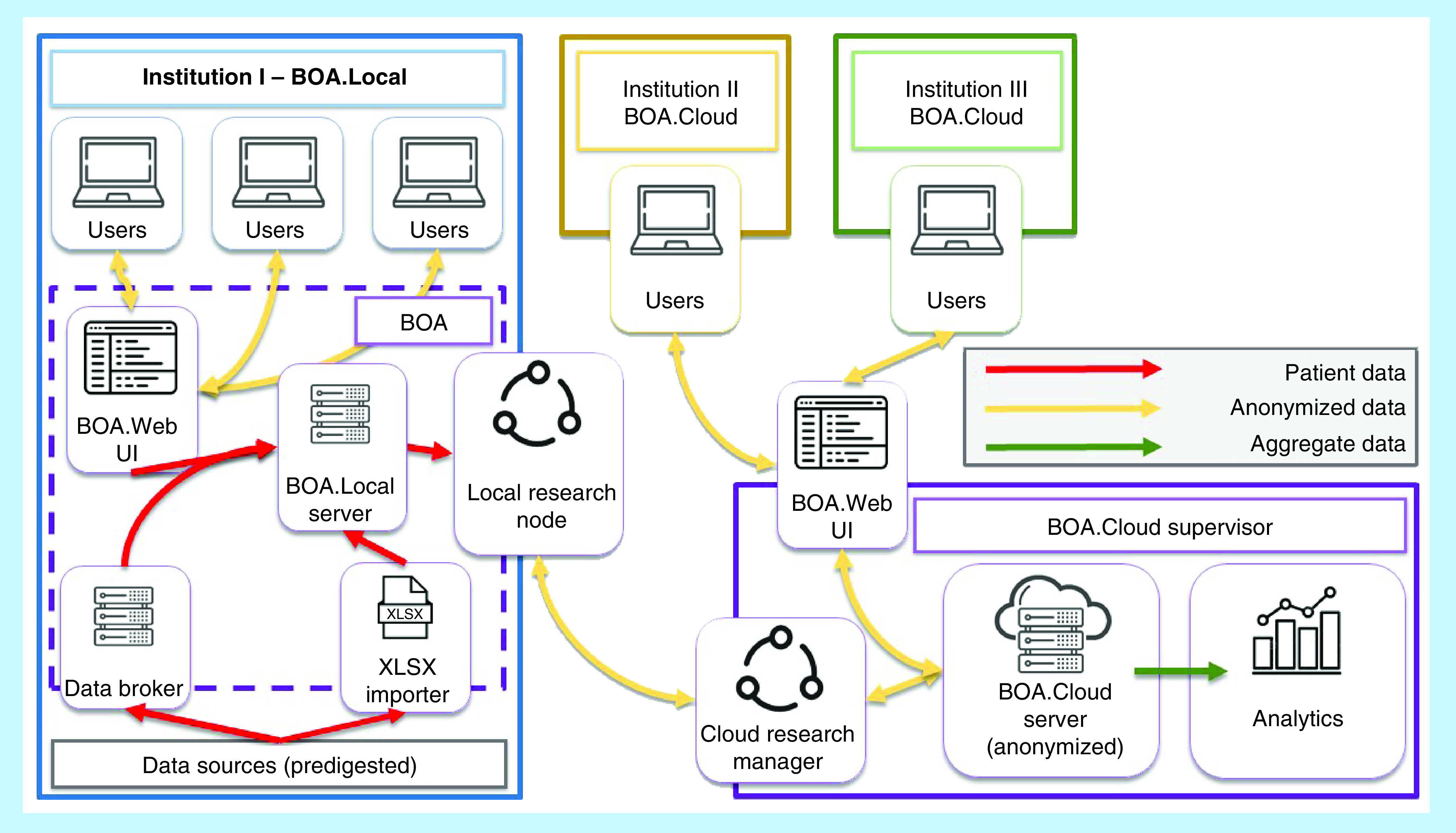
general overview of how the Beyond Ontology Awareness service is laid out. BOA: Beyond ontology awareness.

The two distinct pathways and their optional convergence toward a final database are also highlighted in Figure 3, which depicts a general overview of how the BOA service is laid out. In this particular example, the institution marked with the blue color, works through a BOA.Local installation that can (if desired) upload data to the purple BOA.Cloud Master server installation on demand, while both the institutions marked with the green and yellow colors connect directly to the aforementioned BOA.Cloud Master server, without the need to locally store data.

### Data analysis

One of the goals of the PRE.M.I.S.E. project is to also be able to support multicentric clinical studies. To face this challenge, the project can not rely only on a collection of data stemming from a local repository or a centralized database, as these options present remarkable problems concerning the patient’s privacy. Techniques such as anonymization or de-identification are dangerous because part of the information is shared; data encryption or homomorphic encryption are suboptimal as they can potentially be decrypted. To ensure the patient’s privacy and guarantee data ownership, PRE.M.I.S.E. exploits distributed learning to generate statistical models through multiple separated databases in the various BOA installation sites (both BOA.Local and BOA.Cloud infrastructures): through this paradigm only, aggregated data are shared or transferred – the data never leaves the databases [11] and the metaphorical walls of the institutions that the data is stored in.

In more detail, the distributed learning architecture is composed of one central master node and many client nodes (called local learning nodes) distributed in all database end points. The master node will have the primary task of coordinating and overviewing the learning protocols between the single hospitals, and as such, will never have direct access to clinical data but will only process aggregate data, as necessary for the algorithms intended to be run. The second part of the architecture is composed of the many local learning nodes, which are installed at each hospital. They have access to the local data and perform learning tasks as instructed by the master node. Patient data is not shared with the outside world.

The complete algorithm can be summed up by the following steps:

- The local application learns a model from local data.
- This local model is sent to the master, where it is processed and compared with the models sent by the other hospitals.
- A consensus model is generated and sent back to each hospital for refinement.
- After preset convergence criteria are met, a final consensus model is generated.

The information exchanged between master and local nodes is limited to aggregate values (e.g., parameter weights, general statistics, coefficients) and contains no patient data. All traffic between master and local nodes is managed, monitored and audited by the infrastructure. An entire learning run is an iterative process that usually requires many cycles until the master determines that the learning process has been completed.

In the distributed learning mode, distributed research nodes do not move data around at all: they only apply iterative algorithms that the distributed research master will use to build a consensus model and estimate the model's parameters. Distributed learning can support many algorithms for data analysis. It has been widely used as an inferential regression analysis tools, mainly based on the relationship between outcomes (binary, continuous or multinomial) and covariates, or elements in the dataset. It establishes a data-to-outcome one-way link, investigated using traditional statistical tools as linear models, generalized linear models, survival models and support vector machines [11–13], among others.

The final model can then be presented to the end-user in a variety of ways, such as nomograms, or via interactive websites. In order to become a reliable tool to be used in clinical contexts, each model must undergo a strict evaluation process, mainly based on internal and external validation [14]. Discrimination will be assessed using the c-statistic or area under the curve of the receiver-operating characteristic. The c-statistic is comparable to the area under the curve for dichotomous outcomes but can also be used for Cox regression analyses. Plotting the expected versus the observed outcomes will provide a graphical assessment of the calibration. In addition, to identify variables to be inserted in the ontology, validate variables and build a system that defines variables’ characteristics and relationships among them, the Hosmer–Lemeshow test will be used. The future development of the sharing platform is to involve other radiotherapy centers to combine multiple datasets.

## Results

The ‘umbrella protocol’ has been utilized in order to standardize both the data and procedures, this led to the creation of a consistent dataset reporting ‘personalized treatment registry’, which is paving the way to obtain a trustful analysis for the DSS. A well-defined data collection model – able to collect, standardize and organize features – called ‘Ontology’ was then created.

The team identified more than 130 variables related with brain SRT. All features were collected and organized into three levels: ‘Registry level’, containing epidemiological information; ‘Procedural level’, which includes elements about treatment, toxicities and outcome evaluation and ‘Research level’ where dimensional data, such as imaging information, are collected (Table 1). When identifying variables on a specific technique, attention must be kept on treatment characteristics for every single phase, from the simulation CT scan, to the delivery. We decided to start by grouping treatment variables into three separate categories: ‘contouring’, ‘planning’ and ‘delivery’. We then tackled features related to patient’s set up and subsequently organized different aspects like contouring guidelines to be followed, varying imaging characteristics, clinical target volume, planning target volume margins and lesion(s) localization. In particular, regarding the planning phase, we considered the isodose line prescription, the conformity index, the calculation algorithms, the resolution grid, the multi-lesion treatments with a single isocenter, the beam energy, the gradient index and the normalization method. For the delivery phase, we identified other variables describing image guided radiation therapy techniques (Table 2).

**Table 1.** Extract from brain stereotactic radiotherapy ontology registry level.

Extract from Brain SRT Ontology registry level		
Variables	Definition	Measurement
The phase	The phase of oncologic history in which the patient is evaluated	0: at diagnosis 1: at follow-up 2: at progression or recurrence 3: others missing data
Intent	• Curative: patient who can have a radical treatment • Local control: patient who can benefit of treatment with a stable disease • Palliative: patient with symptoms that can benefit from the treatment or can benefit in terms of quality of life	0: curative 1: local control 2: palliative missing data
Comorbidities	(CharlsonComorbidity Index) (total of the achieved score -> calculate automatically) ACE-27 COMORBIDITY SCORING http://www.rtog.org/LinkClick.aspx?fileticket=oClaTCMufRA%3D&ta	0: no 1: yes/specify (calculate automatically) missing data
Previous oncological history	Site	Specify
	Treatment	0: no 1: yes (if yes, complete relative fields) missing data
	State of previous disease (according to RECIST criteria; if not applicable, refer to specific disease ontology) http://www.eortc.org/investigators-area/recist	0: NED 1: stable complete response 2: stable partial response 3: progression disease missing data

NED: No evidence of disease; SRT: Stereotactic radiotherapy.

**Table 2.** Radiotherapy treatment characteristics.

Set-up		
Variables	Definition	Measurement
RT treatment position		1: supine 2: other (specify)
RT immobilization		0: none 1: thermoplastic masks 3: stereotactic system (true-point-arc?) 4: OSMS 5: frame with bite block and head stabilizer 6: stereotactic helmet 4: others (specify) 999: missing data
Simulation CT scan:	Thickness of CT slice	Value (mm)
	FOV	Value
	Cochlea CT scan	0: no 1: yes 999: missing data
**Contouring**		
Reference guidelines	Guidelines to define target volumes	0: RTOG 1: Scoccianti *et al.* ((15)00080-8/pdf">http://www.thegreenjournal.com/article/S0167-8140(15)00080-8/pdf) 2: AIRO (file:///C:/Users/01903418/Downloads/linee%20guida%20snc%202%20OAR.pdf) 2: Gondi *et al.* ((10)03477-2/fulltext">http://www.redjournal.org/article/S0360-3016(10)03477-2/fulltext) 3: Chera *et al.* (https://www.ncbi.nlm.nih.gov/pubmed/19194118) 4: other (specify) 999: missing data
Organs at risk		0: brain 1: brainstem 2: spinal cord 3: pituitary gland 4: chiasm 5: optic nerve right 6: optic nerve left 7: eye ball (right) 8: eye ball (left) 9: lens (right) 10: lens (left) 11: hippocampus (right) 12: hippocampus left 13: cochlea 14: others (specify) 999: missing data
Imaging to define field		1: T1-weighted brain MR with contrast-enhancement 2: T2-weighted brain MR 3: FLAIR-brain MR 4: CT 5: PET-CT 6: PET-MR 6: 3D MR 7: Other (specify) 999: missing data
Treatment volume	Tumour volume in cc	1: value
Treatment volume	GTV or CTV	1. tumor bed 2. residual mass 3. site of disease 4. others
GTV-CTV margin		mm
CTV-PTV margin		mm
Type of expansion from CTV to PTV		0: isotropic 1: anisotropic 999: missing data
Margin value		0: none mm 1: anterior mm 2: posterior mm 3: lower mm 4: upper mm 5: right mm 6: left mm 999: missing data
Distance between lesions	Distance between two equivalent spheres	$\sqrt{{\left(x2-x1\right)}^{2}+\;{\left(y2-y1\right)}^{2}\;+\;{\left(z2-z1\right)}^{2}-\;}\left(REq2+REq1\right)$
**Prescription**		
**Prescription**	Prescription for all CTVs (a CTV can contain more lesions)	1: dose per fraction (Gy) 2: value of fractions 3: total dose (Gy) 4: CTV description
**Planning**		
RT technique		1: 3D 2: IMRT (sliding window) 3: IMRT (step & shoot) 4: Arc (VMAT) 5: VMAT-SIB 6: IMRT-SIB 7: D-conformed arches MLC 8: archtherapy with cones 9: others 999: missing data
Type of beam		1: photons 2: heavy particles (specify) 999: missing data
Beam energy		1: energy 2: FF 3: FFF 4: dose rate 999: missing data
Geometry isocenters		1: number of isocenters 2: number of of lesions for isocenters 3: distance between lesions with the same isocenter 999: missing data
Geometry beams	Report export	1: number of beams 2: coplanar beams 3: noncoplanar beams 4: number of archs 5: coplanar archs 6: noncoplanar archs 7: complete arch 8: Partial arch 9: collimator angle 999: missing data
Distribution dose		1: homogeneous 2: inhomogeneous 999: missing data
Gradient index		Value
Conformity index		Value
Guidelines		1: TG101 (https://aapm.onlinelibrary.wiley.com/doi/pdf/10.1118/1.3438081) 2: ICRU 91 (https://www.icrp.org/docs/ICRU%20RELEASES%20REPORT%20NO.%2091.pdf) 3: Grimm 2011 (Grimm J, *J. Appl. Clin. Med. Phys.* 12, 267–292 [2011]) 4: Timmerman 2009 (http://mr.crossref.org/iPage?doi=10.3322%2Fcaac.20013) 5: Seminars of Radiation Oncology 2016 (Grimm J, *Semin. Radiation Oncol.* 26, 165–171 [2016])
Method of normalization		0: ICRU point 1: Dmax 2: Isocenter 3: target mean 4: isodose (specify) 5: others 999: missing data
Prescription isodose		Value
TPS version		Specify
Algorithm		Specify
Grid		Specify
Dosimetric parameters		DVH export (research levels)
**Delivery**		
Treatment device		1: model 2: version 3: width lamella-isocenter
Set-up		1: 6 DOF 1: 4 DOF
RT IGRT technique		1: MV-MV 2: MV-KV 3: KV-KV 4: CBCT 5: MR 6: OSMS
RT IGRT frequency		Value
Date of start RT		Date
Date of last day		Value
Elapsed days	For each treatment plane	Value
RT total prescribed dose to PTVs		Value
RT total delivered dose to PTVs		Value

CBCT: Cone beam computed tomography; CT: Computed tomography; CTV: Clinical target volume; DOF: Degrees of freedom; DVH: Dose volume histogram; FF: Flattering filter; FFF: Flattering filter free; FOV: Field of view; GTV: Gross tumour volume; IGRT: Image-guided radiation therapy; IMRT: Intensity-modulated radiation therapy; KV: Kilovoltage; MLC: Multileaf collimator; MR: Magnetic resonance; MV: Megavolts; OSMS: Optical Surface Monitoring System; PTV: Planning treatment volume; RT: Radiation therapy; SIB: Simultaneous integrated boost; TPS: Treatment Planning Systems; VMAT: Volumetric modulated arc therapy.

**Figure 4.**
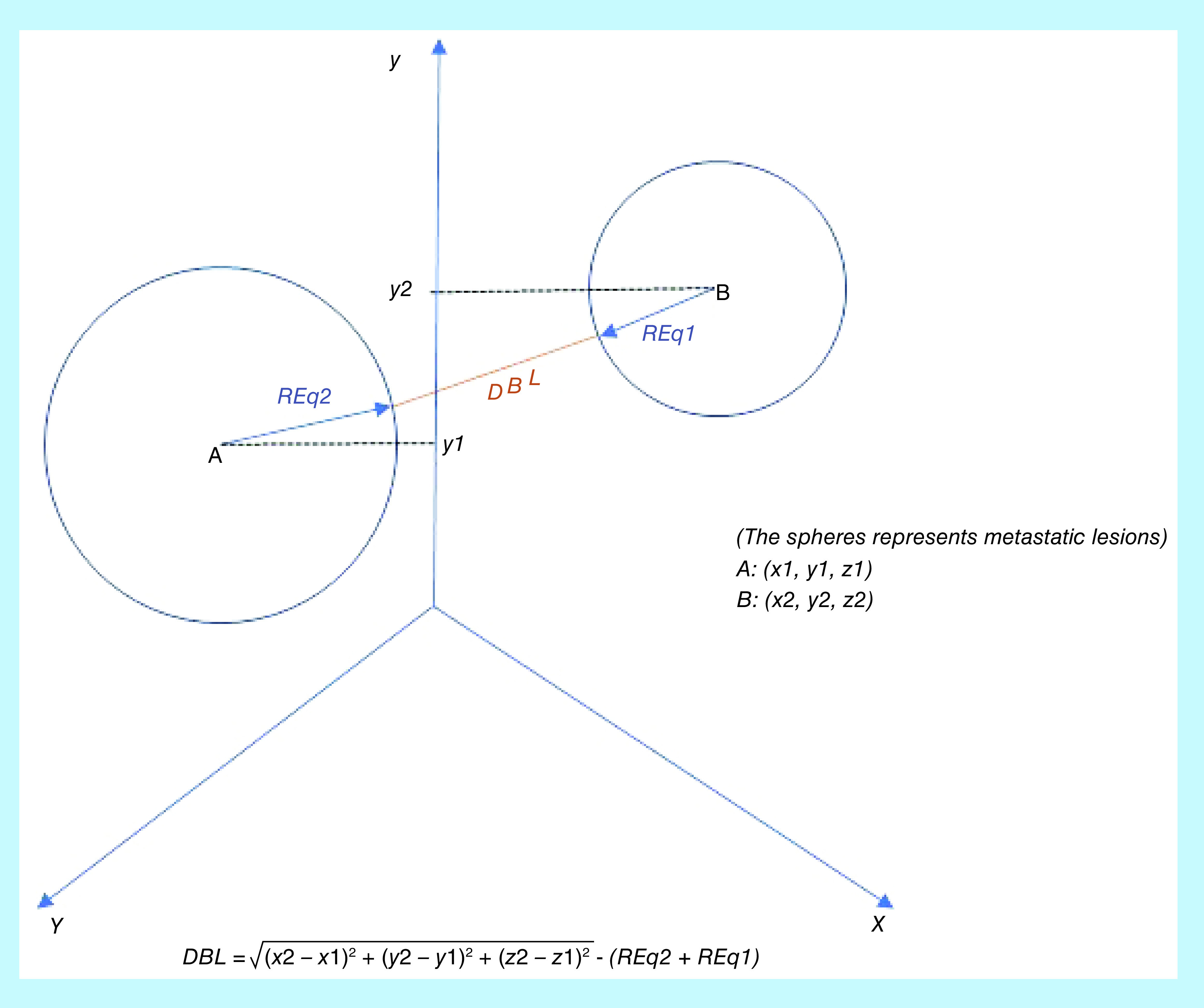
Distance between lesions, equating every lesion to an equivalent sphere.

While the ontology was put into writing, the group realized that one of the most relevant aspects regarding brain metastases is that they are often multiple and the possibility to treat them together with a single isocenter and a single plan depends on their location in the brain. For this reason, in order to easily calculate distance among lesions, we decided to equate every lesion to an equivalent sphere (Figure 4). The BOA web service platform was completely set up and configured for both the BOA.Local and BOA.Cloud pathways, and the ontology has been successfully uploaded. A first institution is currently collecting data using the local server, allowing the institution to store all data without complete anonymization in accordance with the previously reported principles, while a second institution is collecting data through the cloud server. Both involved centers are using ontology-driven CRF [15–17] and all collected data is now available on an on-demand basis, ready to be further processed.

## Discussion

Stereotactic radiosurgery is a radiation therapy technique in which multiple focused radiation beams intersect over a target to deliver a conformal, high-dose radiation and minimal radiation to surrounding normal tissues, thanks to the steep dose gradient. It is usually delivered in a single fraction but can sometimes be delivered over multiple once-daily fractions, usually to a maximum of five [18].

To our knowledge, no standardized data collection system or predictive model focusing on a treatment technique is available in literature. Several ontologies focusing on pathologies and different anatomical sites (e.g., rectum, thyroid and prostate) [1,8,15] can be found in the literature; however, to date, none of these focus on a specific radiotherapy technique (Table 3). Only the brachytherapy ontology can be considered a technique-specific tool but is for head and neck cancers only [4,19].

**Table 3.** Examples of interactive DSS currently in use in clinical practice.

Institution	Ref.
EORTC	[20]
MSKCC	[21]
Dana Farber Cancer Institute and Johns Hopkins Sidney Kimmel Comprehensive Cancer Center	[22]
MGH	[23]
Cancer Research UK	[24]
NCI	[25]
Maastro Clinic	[26]
Policlinico A. Gemelli	[4,19]
Policlinico A. Gemelli	[15]

DSS: Decision support systems; EORTC: European Organization for Research and Treatment of Cancer; MGH: Massachusetts General Hospital; MSKCC: Memorial Sloan Kettering Cancer Center; NCI: National Cancer Institute.

Modified with permission from [16].

The PRE.M.I.S.E. project aims to focus on SRT in every anatomic site, which will lead us to strongly emphasize both the technical and dosimetrical aspects of stereotactic treatments in our ontology. In fact, when approaching SRT from an ontology perspective, a large number of variables have to be taken into consideration. Gantry-based LINAC (lin[ear] ac[celerator]) systems use either fixed circular collimators or multileaf collimators. Treatment planning imaging is based on CT scans, but other images including magnetic resonance images and positron emission tomography, which can be fused to the treatment CT. Once again, different on-board imaging can be used to assure patient alignment. The treatment can be delivered as either multiple arcs or as one continuous arc. The isocenter is generally in the middle of the target lesion, but newer systems with volumetric modulated arc therapy allow for treatment of multiple lesions in a single arc. Dose prescription varies and treatments can be prescribed to the 60–80% isodose line or to 95–98% of the planning target volume and dose distribution can be inhomogeneous or homogenous [27].

In order to easily face the complexity of such a sophisticated technique we decided to start writing the SRT ontology, focusing on brain stereotactic treatment. The choice to build an ontology for each anatomic site was driven both by the need to reduce the risk to deal with a lot of variables, thus omitting relevant ones and by the necessity to reduce the bias of target motion in other anatomic sites (e.g., the lungs and liver).

The team identified more than 130 variables related to brain SRT (isodose line prescription, resolution grid etc.) and organized them into three levels (registry, procedural and research) in order to classify all the information to easily address the query depending on requests. In trying to create an SRT common language we faced a lot of difficulties (Table 4). First, the lack of a unique definition for SRT in terms of dose, fractions and dose homogeneity and second, differences in treatment and planning modalities among different centers, led our research toward collecting a greater number of variables that needed to be included in our ontology, in order to make it suitable for anyone. Another important aspect we faced is represented by multiple lesions treatments. In these cases, lesions can either be treated as a group with a single isocenter or as a single lesion separated from the others, thus, using multiple isocenters. When defining variables for the lesions’ position, we realized that no standard exists in literature for defining tridimensional distance between lesions. We decided to assume each lesion as a sphere and calculate both the distance between the equivalent spheres and the distance among their longitudinal axis. The latter parameter appears to be important in clinical practice when deciding to treat different lesions with single or multiple radiotherapy plans. Collecting the distance between lesions could also be useful because the predictive model could be able to suggest how to treat multiple lesions (with one or multiple isocentres).

**Table 4.** Expected data quality issues and measures of mitigation.

Problem	Example problem	Mitigation	Example mitigation
Completely missing data	Hospital A does not have a diffusion MRI, so all MRI diffusion weighted images derived features are missing. Hospital B has and uses a diffusion MRI in patients with brain metastasis	Impute based on populations from other centers and what is known for the patient	Suppose a (probabilistic) relationship between tumor size and is learned from Hospital B, then the tumor size of Hospital A can be used to infer MRI diffusion weighted parameters in Hospital A even if they don't have a MRI diffusion and are using the same scan protocols
Randomly missing data	Random physician in Hospital A forgets to note the TNM stage of the patient	Because data are missing randomly, the percentage of missing data is generally low and samples are large, machine learning techniques will be unaffected by these errors	Do nothing
Biased data: continuous	An MRI scanner is calibrated differently in Hospital A than in Hospital B, so the SUV values are different	Assuming patients are similar a conversion is possible between two distributions	Determine the distribution of MRI features in Hospital A and B and derive a conversion function from SUVs in Hospital A to hospital B
Biased data: scoring system	CTCAE v3 was used, but after a certain date CTCAE v4 was used to score toxicities	Impute the new score from the old score, if possible	A (probabilistic/deterministic) conversion between the two CTC systems is possible
Random errors	In Hospital A, a physician has noted an incorrect stage on an individual patient	Because errors are random, the percentage errors will be low and samples are large, the effect when using machine learning will be low	Do nothing
Biased missing data	In Hospital A, severe toxicities are noted but mild toxicities are not. In Hospital B, toxicities are always noted		Compare occurrence of toxicities in Hospital A with Hospital B. Detect too low, unexplained mild toxicities in Hospital A. Infer a probability of mild toxicity for patients of Hospital A based on the distribution of Hospital B

CTCAE: Common terminology criteria for adverse events; SUV: Standardized uptake value; TNM: Tumor, node, metastasis.

Brain SRT is usually employed when treating brain metastases. This aspect implies the need to include the primary tumor and its stage in the ontology not excluding all complementary treatments. We considered variables regarding new therapies such as immunotherapy and target therapy, for which an internationally recognized standard timing for concomitant radiotherapy is not yet available.

PRE.M.I.S.E. perspectives reside in the need to develop a system allowing the clinical decision-making process to be shared between physicians and patients in order to choose the best tailored treatment.

This project could lead to the development of predictive models based on individual patients features complementing the existing consensus or guidelines. The large amount of clinical data can then be further processed either through more classical statistical approaches or through the use of modern machine learning tools, which can be further refined into reliable clinical decision making support tools in order to guarantee a personalized approach to medicine. Clinical evidences are difficult to be generated rapidly, in a reliable way and the analysis of retrospective case series can present data collection biases due to known outcomes.

## Conclusion

This project represents the first example of a standardized data collection system created for a particular radiation therapy technique and specifically for SRT. The next step of this initiative is patient enrollment. The setup of a DSS-based validated prediction model represents the long term aim of the project and could be helpful in personalizing treatment choices, both in terms of efficacy and toxicity and in identifying the most suitable patients to be included in future randomized clinical trials [8,28].

## Future perspective

We intend to substantially expand the number institutions engaged with the project and the data collection efforts, initiating a parallel effort to incorporate start an ontology for stereotactic body radiation therapy into the workflow. Moreover, we aim to provide a DSS capable of individualizing the SRT treatment: developing, validating and improving prediction models for overall survival, local control, disease free survival as well as acute and late radiation-induced side effects relevant for patients that undergo a stereotactic treatment.

These prediction models could be very useful to better informs patients on the risks (acute and late toxicity) and benefits of the treatment.

**Summary points**

- ‘Personalized medicine’ is defined by the National Cancer Institute (MA, USA) as a “form of medicine that uses information about a person’s genes, proteins and environment to prevent, diagnose and treat disease. In cancer, personalized medicine uses specific information about a person’s tumor to help diagnose, plan treatment, find out how well treatment is working or make a prognosis”.
- The tendency toward individualized medicine and the increasing amount and complexity of data, makes extremely difficult to identify which clinical decisions are better for specific patients.
- In daily clinical practice, decision support systems could help to personalize clinical choice.

**Ontology levels**

- The ontology is a system to collect heterogeneous data in a standardized way in order to create large databases.
- The creation of an ontology increased the power of description by moving from local data dictionaries to a global data vocabulary.

**Storage system level**

- The storage system architecture is based on the use of a specific software called Beyond Ontology Awareness, which proposes two distinct data consolidation approaches and two data processing strategies.

**Distributed learning**

- The complete algorithm can be summed up by the following steps:
  - The local application learns a model from local data;
  - This local model is sent to the master, where it is processed and compared with the models sent by the other hospitals;
  - A consensus model is generated and sent back to each hospital for refinement;
  - After preset convergence criteria are met, a final consensus model is generated.
- PRE.M.I.S.E. project innovation resides mainly in having created an ontology for a particular radiation therapy technique instead of creating a model that only concerns a specific pathology.

**Future perspective**

- To provide decision support system capable of individualizing the treatment:
  - Development, validation and improvement of prediction models for overall survival, local control and disease-free survival for patients that undergo a stereotactic treatment;
  - Development, validation and improvement of prediction models for acute and late radiation-induced side effects relevant for patients that undergo a stereotactic treatment;
  - Use of prediction models to better informs patients on the risks (acute and late toxicity) and benefits of the treatment.

## References

[1] Alitto A, Gatta R, Vanneste B PRODIGE: prediction models in prostate cancer for personalized medicine challenge. Future Oncol. 13(24), 2171–2181 (2017). 28758431 10.2217/fon-2017-0142

[2] Valentini V, Dinapoli N, Damiani A The future of predictive models in radiation oncology: from extensive data mining to reliable modeling of the results. Future Oncol. 9(3), 311–313 (2013). 23469966 10.2217/fon.12.197

[3] Booth CM, Tannock IF Randomised controlled trials and population-based observational research: partners in the evolution of medical evidence. Br. J. Cancer 110(3), 551–555 (2014). 24495873 10.1038/bjc.2013.725 PMC3915111

[4] Tagliaferri L, Kovács G, Autorino R ENT COBRA (Consortium for Brachytherapy Data Analysis): interdisciplinary standardized data collection system for head and neck patients treated with interventional radiotherapy (brachytherapy). J. Contemp. Brachytherapy 8(4), 336–343 (2016). 27648088 10.5114/jcb.2016.61958 PMC5018530

[5] Gruber TR A translation approach to portable ontology specifications. Knowledge Acquisition 5(2), 199–220 (1993).

[6] Valentini V, van Stiphout RGPM, Lammering G Nomograms for predicting local recurrence, distant metastases, and overall survival for patients with locally advanced rectal cancer on the basis of European randomized clinical trials. J. Clin. Oncol. 29(23), 3163–3172 (2011). 21747092 10.1200/JCO.2010.33.1595

[7] Gorlia T, van den Bent MJ, Hegi ME Nomograms for predicting survival of patients with newly diagnosed glioblastoma: prognostic factor analysis of EORTC and NCIC trial 26981-22981/CE.3. Lancet Oncol. 9(1), 29–38 (2008). 18082451 10.1016/S1470-2045(07)70384-4

[8] Tagliaferri L, Gobitti C, Colloca GF A new standardized data collection system for interdisciplinary thyroid cancer management: thyroid COBRA. Eur. J. Intern. Med. 53, 73–78 (2018). 29477755 10.1016/j.ejim.2018.02.012

[9] Meldolesi E, van Soest J, Dinapoli N An umbrella protocol for standardized data collection (SDC) in rectal cancer: a prospective uniform naming and procedure convention to support personalized medicine. Radiother. Oncol. 112(1), 59–62 (2014). 24853366 10.1016/j.radonc.2014.04.008

[10] Traverso A, van Soest J, Wee L, Dekker A The radiation oncology ontology (ROO): publishing linked data in radiation oncology using semantic web and ontology techniques. Med. Phys. 45(10), 854–862 (2018). 10.1002/mp.12879 30144092

[11] Damiani A, Vallati M, Gatta R Distributed learning to protect privacy in multi-centric clinical studies. : Lecture Notes in Computer Science. Holmes JH, Bellazzi R, Sacchi L, Peek N (). Springer-Verlag, NY, USA, 65–75 (2015).

[12] Boyd S, Parikh N, Chu E, Peleato B, Eckstein J Distributed optimization and statistical learning via the alternating direction method of multipliers. Foundations Trends Machine Learning. 3(1), 1–122 (2010).

[13] Lu CL, Wang S, Ji Z Web DISCO: a web service for distributed cox model learning without patient-level data sharing. J. Am. Med. Inform. Assoc. 22(6), 1212–1219 (2015). 26159465 10.1093/jamia/ocv083 PMC5009917

[14] Harrell F Resampling, validating, describing, and simplifying the model. Regression Modeling Strategies. Springer-Verlag, NY, USA (2001).

[15] Meldolesi E, van Soest J, Alitto AR VATE: validation of high technology based on large database analysis by learning machine. Colorectal Cancer 3(5), 435–450 (2014).

[16] Meldolesi E, van Soest J, Damiani A Standardized data collection to build prediction models in oncology: a prototype for rectal cancer. Future Oncol. 12(1), 119–136 (2016). 26674745 10.2217/fon.15.295

[17] Meldolesi E, van Soest J, Dinapoli N An umbrella protocol for standardized data collection (SDC) in rectal cancer: a prospective uniform naming and procedure convention to support personalized medicine. Radiother. Oncol. 112(1), 59–62 (2014). 24853366 10.1016/j.radonc.2014.04.008

[18] Badiyan SN, Regine WF, Mehta M Stereotactic radiosurgery for treatment of brain metastases. J. Oncol. Pract. 12(8), 703–712 (2016). 27511715 10.1200/JOP.2016.012922

[19] Tagliaferri L, Budrukkar A, Lenkowicz J Review papers ENT COBRA ONTOLOGY: the covariates classification system proposed by the Head & Neck and Skin GEC-ESTRO Working Group for interdisciplinary standardized data collection in head and neck patient cohorts treated with interventional radiotherapy (brachytherapy). J. Contemp. Brachytherapy 10(3), 260–266 (2018). 30038647 10.5114/jcb.2018.76982 PMC6052377

[20] Prognostic Calculators (2020). www.eortc.org http://www.eortc.be/tools/lggcalculator/

[21] Prediction tools – A tool for doctors and patients (2020). www.mskcc.org/nomograms

[22] BayesMendel Lab (2020). http://bcb.dfci.harvard.edu

[23] LifeMath (2020). http://www.lifemath.net

[24] Cancer Risk Assessment tool (RAT) (2020). www.cancerresearchuk.org

[25] Cancer risk prediction and assessment (2020). http://epi.grants.cancer.gov

[26] MAASTRO (2020). www.predictcancer.org

[27] Halasz L, Rockhill J Stereotactic radiosurgery and stereotactic radiotherapy for brain metastases. Surg. Neurol. Int. 4(5), 185 (2013). 10.4103/2152-7806.111295 PMC3656557 23717789

[28] Skripcak T, Belka C, Bosch W Creating a data exchange strategy for radiotherapy research: towards federated databases and anonymised public datasets. Radiother. Oncol. 113(3), 303–309 (2014). 25458128 10.1016/j.radonc.2014.10.001 PMC4648243

